# Degradable Cross-Linked Nanoassemblies as Drug Carriers for Heat Shock Protein 90 Inhibitor 17-*N*-Allylamino-17-demethoxy-geldanamycin

**DOI:** 10.3390/ph4101281

**Published:** 2011-09-26

**Authors:** Andrei Ponta, Shanjida Akter, Younsoo Bae

**Affiliations:** Department of Pharmaceutical Sciences, College of Pharmacy, University of Kentucky, 789 South Limestone, Lexington, KY 40536-0596, USA

**Keywords:** drug delivery, controlled drug release, nanoassemblies, heat shock protein 90, cancer chemotherapy

## Abstract

Cross-linked nanoassemblies (CNAs) with a degradable core were prepared for sustained release of 17-N-allylamino-17-demethoxygeldanamycin (17-AAG), a potent inhibitor of heat shock protein 90 (HSP90). The particle size of CNAs ranged between 100 and 250 nm, which changed depending on the cross-linking yields and drug entrapment method. CNAs with a 1% cross-linking yield entrapped 17-AAG in aqueous solutions, yet degraded in 3 hrs. CNAs entrapped 5.2 weight% of 17-AAG as the cross-linking yield increased to 10%, retaining more than 80% of particles for 24 hrs. CNAs with drugs entrapped after the cross-linking reactions were 100 nm and remained stable in both pH 7.4 and 5.0, corresponding to the physiological, tumoral, and intracellular environments. Drug was completely released from CNAs in 48 hrs, which would potentially maximize drug delivery and release efficiency within tumor tissues. Drug release patterns were not negatively affected by changing the cross-linking yields of CNAs. CNAs entrapping 17-AAG suppressed the growth of human non-small cell lung cancer A549 cells as equally effective as free drugs. The results demonstrated that CNAs would be a promising formulation that can be used in aqueous solutions for controlled delivery and release of 17-AAG.

## 1. Introduction

A heat shock protein (HSP) is a molecular chaperone that refolds damaged proteins in the cell [1,2]. Protein refolding is responsible for cell survival under stressed conditions, and most cancer cells take advantage of the HSP-mediated cell protection machinery by overexpressing HSPs [3]. HSPs are involved in refolding more than 300 types of proteins, often called client proteins [4], which include not only essential proteins used for the cellular signaling cascade but also all six hallmarks of cancer (self-sufficient growth, insensitivity to anti-growth, tissue invasion, limitless reproduction, sustained angiogenesis, and apoptosis evasion).

HSPs work in the cell as complexes, and different family members are involved [5,6]. Among HSPs, 90 kDa HSP (HSP90) plays an important role in regulating the complex formation and subsequent biological action [7]. In an effort to stop HSP functions selectively in cancer cells, a large number of HSP90 inhibitors have been developed. 17-N-Allylamino-17-demethoxygeldanamycin (17-AAG) is one of the promising HSP90 inhibitors, derivatized from a benzoquinone ansamycin antibiotic geldanamycin [8]. 17-AAG is effective in the low nanomolar concentration range in suppressing cancer cell growth. It is less toxic than geldanamycin, a chemotherapeutic agent that failed preclinical trials due to serious hepatotoxicity [9]. Low water-solubility and poor bioavailability are two major issues that limit clinical applications of 17-AAG [10].

Drug delivery systems using nanoparticles (nanoparticulate drug delivery) have drawn attention in recent years as technology that improves water-solubility, tumor preferential accumulation, and controlled release of anticancer drugs [11-13]. Most anticancer drugs are highly toxic and hardly soluble in aqueous solutions. Solubilizers, such as DMSO, Cremophor EL and other surfactants, are frequently used to prepare drug formulations, yet these formulations often cause adverse effects in cancer patients (skin reactions, headache, nausea, vomiting, diarrhea, and allergic reactions) [14].

**Figure 1:**
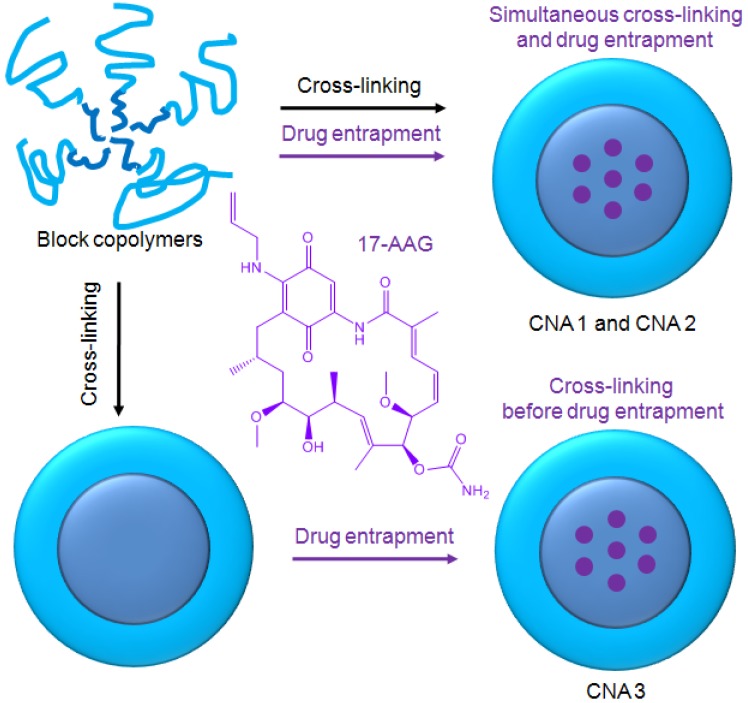
Preparation of CNAs entrapping 17-AAG.

Polymer micelles are one of the most promising drug carriers used for nanoparticulate drug delivery. They are being tested in multiple clinical trials in several countries [15,16]. We have been developing multifunctional polymer micelles to enhance therapeutic efficacy and reduce toxicity of various therapeutic agents [17-23]. Polymer micelles are nanoscale particles (< 200 nm) comprising a hydrophobic core enveloped by a hydrophilic shell. The hydrophobic core acts as a nano compartment for drug loading while the hydrophilic shell improves the particle solubility and prevents protein adsorption that triggers the immune response in the body. Our previous studies demonstrated that polymer micelles carried anticancer drugs to tumor tissues preferentially, reducing toxicity in animal tumor models [19]. Polymer micelles appeared to be a versatile platform for geldanamycin analogues alone and in combination with other anticancer drugs [22]. Chemical conjugation between drug molecules was used in these studies to prevent burst drug release and to fine tune the overall drug release patterns by using degradable linkers such as ester and hydrazone bonds [23,24]. In comparison to other cancer drugs, 17-AAG has no functional groups that can be used for chemical conjugation to drug carriers without altering its biological activity (Figure 1). In this study, therefore, we prepared a cross-linked nanoassembly (CNA) that has a degradable core in which 17-AAG can be physically entrapped and preserved stably in aqueous solutions for a prolonged time period. This novel approach is expected to deliver 17-AAG to tumor tissues efficiently without complicated synthesis of drug analogues for chemical conjugation.

**Scheme 1:**
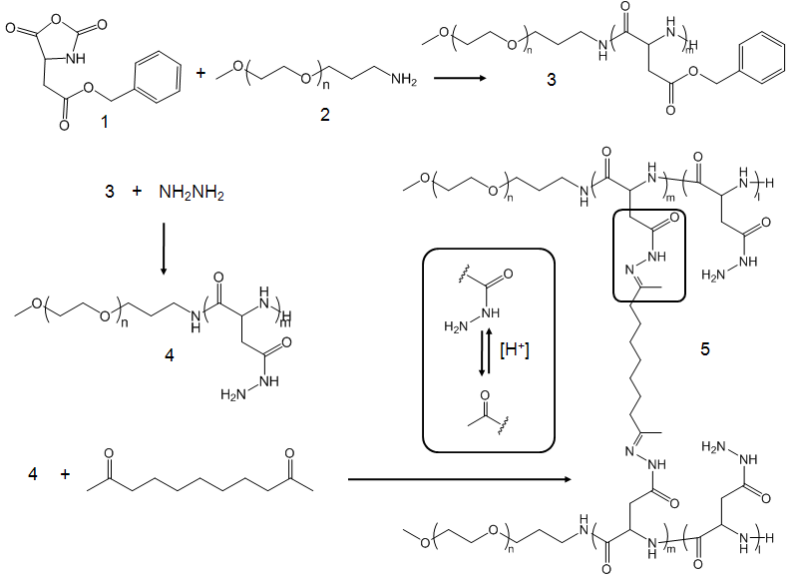
Synthesis of block copolymers and CNAs.

CNAs were previously shown to improve particle stability and entrap drug effectively without changing the particle properties before and after drug entrapment [25]. We prepared CNAs for 17-AAG by cross-linking water soluble poly(ethylene glycol)-poly(aspartate hydrazide) block copolymers with undecanedione, introducing degradable hydrazone cross-linkers (Scheme 1). Drugs were entrapped in the CNAs with different cross-linking ratios during and after the cross-linking reactions. This article reports nanoparticulate properties of CNAs entrapping 17-AAG, which provide a better understanding of this novel drug delivery formulation for its further development in pre-clinical and clinical development.

## 2. Experimental Section

### 2.1. Chemicals and Cell-Line

α-Methoxy-ω-amino poly(ethylene glycol) (PEG, MW = 5,000) was purchased from NOF Corporation (Tokyo, Japan). Anhydrous tetrahydrofuran, anhydrous hexane, anhydrous dimethylsulfoxide (DMSO), anhydrous hydrazine, benzene, anhydrous ethyl ether, L-aspartic acid β-benzyl ester, triphosgene, and 3,9-undecadione were purchased from Sigma-Aldrich Inc. (St. Louis, MO, USA). Regenerated cellulose dialysis bags with 6-8,000 molecular weight cut off (MWCO), Slide-A-Lyzer ^®^ dialysis cassettes with 10,000 MWCO, acetate buffer solution, and phosphate buffer solution were purchased from Fisher Scientific (Pittsburg, PA, USA). 17-(Allylamino)-17-demethoxygeldanamycin (17-AAG) was purchased from LC Laboratories (Woburn, MA, USA). The lung cancer cell line A549, and F12K media were purchased from ATCC (Manassas, VA, USA). Fetal bovine serum (FBS) was purchased from Atlanta Biological (Atlanta, GA, USA).

### 2.2. Cross-Linked Nanoassembly Synthesis

Poly(ethylene glycol)-poly(aspartate hydrazide) [PEG-p(Asp-Hyd)] block copolymers were synthesized as reported previously [18,23] (Scheme 1). Briefly, l-aspartic acid β-benzyl ester was reacted with triphosgene to prepare N-carboxyanhydride (NCA) monomers, which were subsequently polymerized by using PEG as a macroinitiator. Synthesized PEG-poly(β-benzyl l-aspartate) block copolymers were reacted with anhydrous hydrazine to introduce hydrazide groups. Activated block copolymers were purified by ether precipitation, and freeze-dried to collect PEG-p(Asp-Hyd) block copolymers comprising of 5 kDa PEG and 33 repeating units of aspartic acid. Nanoassemblies (CNAs) were subsequently prepared by reacting PEG-p(Asp-Hyd) and 3,9-undeca-dione in DMSO at 50 mg/mL. The reaction was allowed to proceed for two days at 37 °C. The cross-linking degree was aimed at 1 and 10% by adjusting the molar ratios between hydrazide groups of PEG-p(Asp-Hyd) and ketone groups of 3,9-undecanedione CNAs were purified and collected. Samples of CNAs with 10% cross-linking were kept for separately for characterization. Remaining CNAs were used for drug entrapment as described below.

### 2.3. Drug Entrapment

Drug was entrapped in CNAs by two methods. One was to entrap drugs while conducting the block copolymer cross-linking reactions. The other method was to prepare CNAs first and allow the CNAs to absorb drug later. For simultaneous drug entrapment, PEG-p(Asp-Hyd) (200 mg, 21.5 μM) and 17-AAG (50 mg, 85.5 μM) were dissolved in DMSO at 50 mg/mL in a scintillation vial. Undecanedione was separately dissolved in DMSO, and subsequently added to the block copolymer drug mixture. The amount of undecadione was adjusted according to the cross-linking yields (1 or 10%). The reaction was allowed to proceed for two days at 37 °C. The final DMSO solution was diluted using deionized (DI) water in preparation for filtration using a 0.22 μM filter. Immediately after filtration, products were dialyzed for six hours, against DI water using a regenerated cellulose membrane (MWCO 6-8,000). Replacing water frequently led to the removal of free 17-AAG, DMSO, and undecadione. The drug-loaded CNAs were collected after freeze drying from water. CNAs entrapping drugs after cross-linking reactions were prepared by mixing the CNAs and 17-AAG in DMSO. The mixture was kept at 37 °C for two days. The solutions were filtered (0.22 μm) and dialyzed (MWCO 6-8,000). Products were collected by freeze-drying. Drug loading was determined using a SpectraMax M5 (Molecular Devices, Sunnyvale, CA, USA), equipped with SoftMaxPro software. 17-AAG was quantified at 336 nm.

### 2.4. Particle Stability Test

Particle stability of CNAs was tested at pH 7.4 and 5.0 over 48 h periods (0, 1, 3, 6, 24, and 48 hrs). CNAs (2 mg/mL) were dissolved in acetate buffer solution (pH 5.0, 10 mM) or phosphate buffer solution (pH 7.4, 10 mM). Particle size and intensity were observed by a Zetasizer Nano-ZS (Malvern, Worcestershire, UK) equipped with He-Ne laser (4 mW, 633 nm) light source configured with 173° light scattering angle.

### 2.5. Drug Release Experiments

CNA solutions were prepared by dissolving the freeze-dried CNA powders in aqueous solutions at pH 7.4 and 5.0. An initial sample was taken from the solution for the zero hour time point. The remaining CNA solution was split into two equivalent parts and place into Slide-A-Lyzer ^®^ (Thermo Scientific, Rockford, IL, USA) dialysis cassettes (MWCO = 10,000). The cassettes were in turn placed in 4.0 L of 10 mM buffer solutions. Temperature was maintained at a constant 37 °C. At specific intervals (1, 3, 6, 24, and 48 hrs), 150 μL of sample solution was removed from the dialysis cassettes for UV-Vis analysis. Each drug release experiment was repeated three times.

### 2.6. Cytotoxicity Assays

The human non-small cell lung cancer cell line A549 was used to determine the cytotoxicity of 17-AAG alone as well as the CNA formulations. Sample stock solutions were prepared at a concentration of 10 mM with respect to 17-AAG, as confirmed by UV-Vis analysis. Serial dilutions were then prepared with the lowest concentration 10 nM. A549 cells were seeded in a 96 well plate (5,000 cells/well) in 100 μL of F12K media containing 10% FBS. After 24 h, the media were removed, and replaced with drug containing media with a maximum DMSO content of 1%. Cells were kept at 37 °C and 5% CO_2_ for 72 hours. Cell viability was assessed using a resazurin assay that indicates mitochondrial metabolic activity in live cells. 10 μL of a 1 mM resazurin solution in PBS was added to the vehicle- and drug-treated cells at the end of the treatment period. Cell viability was determined three hours later by reading the fluorescence at 560 nm (Ex)/590 nm (Em). The fluorescence signals were quantified using a Spectramax M5 plate reader (Molecular Devices) equipped with a SoftMaxPro software. Cytotoxicity was determined by calculating the half maximal inhibitory concentration (IC50) of each sample with Prism software.

### 2.7. Statistics

Each experiment was performed in at least triplicates. Data were recorded as average ± standard deviation. Microsoft Excel (2007) and Prism software were used for data analysis.

## 3. Results and Discussion

### 3.1. Block Copolymer and CNA Synthesis

The synthesis process of PEG-p(Asp-Hyd) is shown in Scheme 1. Ring opening polymerization of NCA produced PEG-poly(β-benzyl L-aspartate) [PEG-PBLA] block copolymers. Products contained 33 BLA repeating units, which was determined by ^1^H-NMR. This was achieved by comparing the peak areas from PEG (3.5 ppm) and benzyl groups (7.2 ppm). The benzyl groups were completely replaced with hydrazide groups following the aminolysis reaction between PEG-PBLA and hydrazine. The purity of PEG-p(Asp-Hyd) was confirmed as we previously reported [23]. CNAs were prepared by reacting PEG-p(Asp-Hyd) and undecadione. Hydrazide groups on the block copolymers formed an acid-labile hydrazone bond with undecadione. No additional chemicals were used during the cross-linking reaction. Aimed cross-linking degree was adjusted by controlling the amount of undecadione used in the reaction. ^1^H-NMR was used to confirm the insertion of undecanedione. However, potential side-reactions, such as partial conjugation of undecadione and cross-linking within the same block copolymer chain, could not be identified completely in this study.

### 3.2. Particle Size and Drug Entrapment

Table 1 summarizes the particle size and drug entrapment yields of all CNAs prepared in this study. CNAs with 1% cross-linking had an average particle size of 177.6 nm. The particle size distribution of CNAs with 1% cross-linking (CNA 1) was broad, but no distinctive populations of particles were observed. The particle size of CNAs increased to 183.5 nm as the core cross-linking yield became 10% (CNA 2). CNA 2 showed a narrow particle size distribution. Higher cross-linking yields seem to be more effective to tether PEG-p(Asp-Hyd) chains without affecting the particle size. CNA1 entrapped 5.2 wt% of 17-AAG. The drug entrapment yield was increased for CNA 2 to 15.7 wt%. CNA 2 entrapped three times more drugs compared to CNA 1 although the particle size remained the same. The drug entrapment method changed the particle size of CNAs. CNAs to which drugs were entrapped after cross-linking (CNA 3) were smaller than CNA 2. The particle size of CNA 3 was 109.4 nm, which was two-fold smaller than that of CNA 2 where drugs were entrapped during cross-linking. Entrapping drugs in CNA 3 achieved 14.1 wt% drug loading, which was comparable with the drug entrapment yield in CNA 2. Blank CNAs with 10% cross-linking had a particle size of 29.4 nm with a narrow particle size distribution. After drug loading, CNA particle size, irrespective of the cross-linking degree, increased to more than 100 nm. These results suggest that the core of CNAs can still swell even at the 10% cross-linking yield during drug entrapment.

### 3.3. Particle Stability

**Figure 2:**
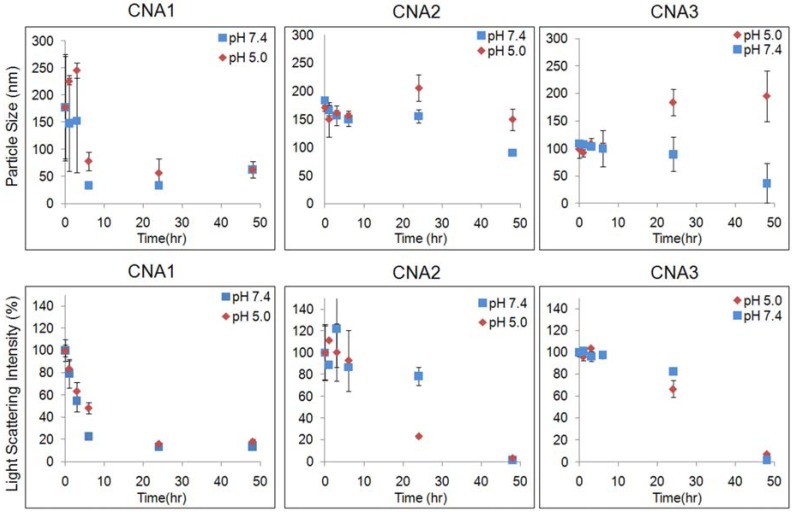
Time-dependent changes in particle sizes and light scattering intensity of CNAs.

Particle stability of CNAs was characterized at pH 7.4 and 5.0, which correspond to the physiological condition (pH 7.4) and the acidic *in vivo* environments such as tumor tissues (pH 6.5) and lysosomes (5< pH < 6.5). CNAs without 17-AAG maintained a particle size of about 30 nm irrespective of pHs. Particle size remained relatively unchanged over a 48 h period in both pH 7.4 and 5.0 (Figure 2). It is noticeable that only CNA3 showed a slight change in particle size in different pHs.

Light scattering intensity was also observed in order to ensure that particles did not degrade over time. Interestingly, at pH 7.4 70% of the initial light scattering was observed after 3 h, but this intensity was maintained until the 48 h time point. At pH 5.0, only 20% of the initial light scattering intensity was lost during the duration of the experiment. It was apparent that CNAs remained present after a 48 h period, at both pH 5.0 and 7.4. The initial particle size of drug loaded CNAs was similar at both pH 7.4 and 5.0, indicating that CNAs can remain stable in the blood and tumor tissues following intravenous injections. CNA 1 degraded rapidly, and only 20% of initial light scattering intensity was observed after 3 hrs. CNA 2 improved the particle stability, retaining more than 80% of light scattering intensity at a constant particle size. Degradation of CNA 2 was accelerated at pH 5.0, while the CNAs remained stable at pH 7.4 up to 24 hrs. When drugs were entrapped in CNAs after cross-linking, particle stability was improved at pH 5.0 as well as 7.4, retaining more than 60% of CNA 3 in the acidic environment. All CNAs degraded completely over 48 h at pH 7.4 and 5.0. These results demonstrate that drug entrapment after preparing empty CNAs is effective to prepare stable CNAs that can preserve drug molecules in physiological and acidic tumor environments for prolonged time.

### 3.4. Drug Release Experiments

**Figure 3:**
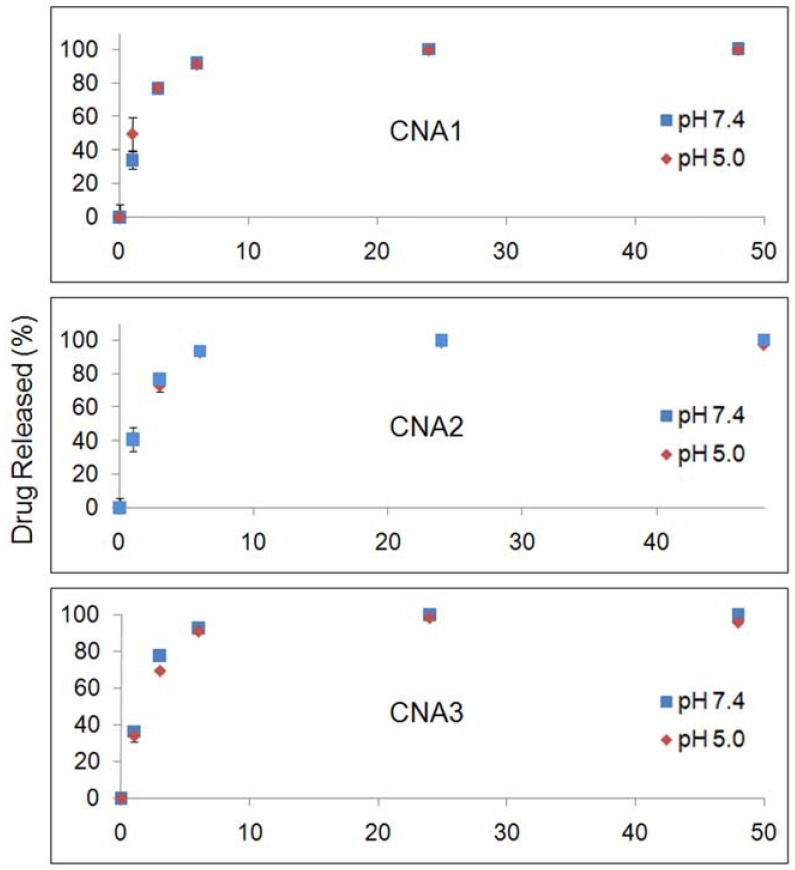
Drug release patterns of CNAs entrapping 17-AAG.

Figure 3 shows drug release patterns from CNAs incubated at pH 7.4 and 5.0. CNA 1 released more than 80% of 17-AAG in 6 hrs. The cross-linking yields did not slow down the drug release from CNAs. CNA 2 and CNA 3 with 10% cross-linking yields) showed a similar drug release pattern in comparison to CNA 1 (1% cross-linking). The drug entrapment method did not change the drug release patterns, neither accelerating nor slowing down drug release at pH 7.4 and 5.0. Particle stability and drug release patterns were inconsistent, because CNA 2 and 3 that remained stable for 24 hrs released drugs as quickly as CNA 1 that degraded in 3 hrs. It is surmised that the cross-linked core of CNAs is leaky enough even at a 10% cross-linking yield for 17-AAG to diffuse through the core easily while the drug molecules strongly interact with the core-forming segment of PEG-p(Asp-Hyd). It must be noted that the sampling at 24 h and 48 h, which both yielded near complete drug release from all CNAs, did not provide further insight in understanding the kinetics of drug release, although the time points in Figures 2 and 3 were matched for drug release and particle stability studies. The release data for free 17-AAG would be also necessary to validate our findings more accurately as suggested by other research groups [26]. However, our attempts were unsuccessful to determine accurate concentrations of free 17-AAG in a dialysis bag due to drug precipitation. Further studies are necessary to elucidate how 17-AAG molecules are present in the core of CNAs.

### 3.5. Cytotoxicity Assays

**Figure 4:**
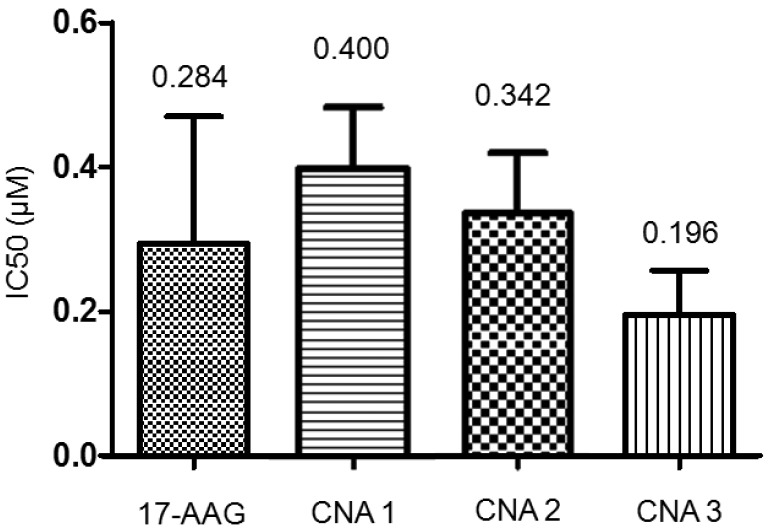
IC_50_ values of CNAs entrapping 17-AAG in A549 cell line.

Biological activity of drug-entrapped CNAs was tested in A549 cell lines, using free 17-AAG in DMSO as a positive control. CNA 2 and 3 had IC_50_ values of 342 nM and 196 nM, respectively, following sample exposure to A549 for 72 hrs (Figure 4).

CNA 1 had an IC_50_ of 400 nM, indicating that cross-linking degree does not affect the activity of 17-AAG. Free 17-AAG showed an IC_50_ value of 284 nM under the sample conditions. A one-way ANOVA revealed that there was no statistical significance among the IC_50_ values of free drug and drug-entrapped CNAs.

**Figure 5:**
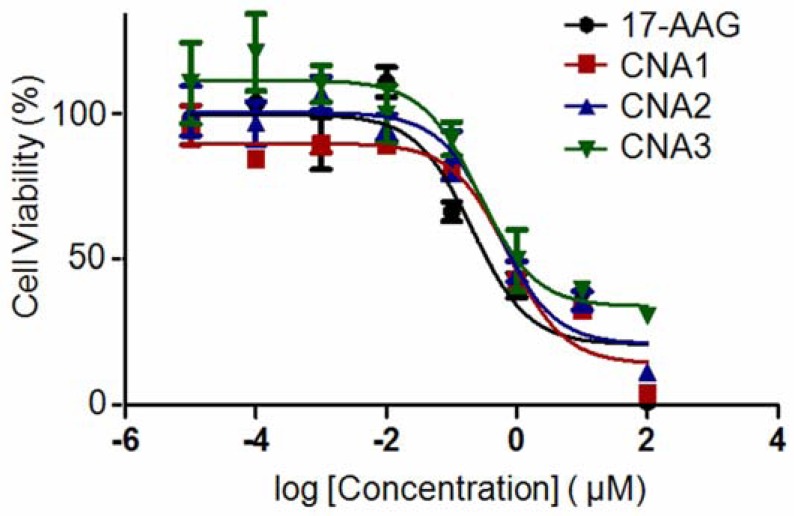
Viability curves of A549 cells exposed to CNAs entrapping 17-AAG.

Interestingly, CNAs with drugs entrapped after cross-linking induced cytostatic effects on the cells, and more than 30% cells were still alive even at 100 μM (Figure 5). Other drug-entrapped CNAs and free 17-AAG caused complete cell death at 100 μM, although the overall viability curves looked similar up to 10 μM. These results suggest that CNA formulations can be used to change biological efficacy of 17-AAG, although further investigation is necessary.

## 4. Conclusions

In this study, degradable CNAs were prepared as a delivery carrier for 17-AAG. The cross-linking yield and drug entrapment method were tested to prepare drug-entrapped CNAs with structural uniformity, particle stability, and drug release patterns relevant to clinical applications. CNAs showed high drug entrapment yields (5.2∼15.7 wt%) and complete drug releases in 48 hrs. CNAs had narrow particle size distribution as the cross-linking yield increased from 1 to 10%. The 10% cross-linking yield appeared to stabilize CNAs without affecting the drug release patterns at pH 7.4 and 5.0. CNAs with drugs entrapped after cross-linking of the core were stable, and more than 60% of CNAs preserved drugs in aqueous solutions at 37 °C for 24 hrs. CNAs released 17-AAG in an active form to cause cell growth inhibition in cancer cells at the drug concentration similar to that of free drugs. Cancer cells showed differential cellular response (cytotoxic and cytostatic) to CNAs depending on the drug entrapment method. Therefore, CNAs are expected to provide an efficient and convenient nanoscale drug delivery vehicle for 17-AAG and other hydrophobic drugs that have issues such as low water solubility, poor bioavailability, and limited drug accumulation in tumors.

## Figures and Tables

**Table 1:** Characterization of CNAs entrapping 17-AAG.

**CNA**	**Particle Size (nm)**		**Drug Loading (wt %)**

	**pH 7.4**	**pH 5.0**	
CNA 1	177.6 ± 94.5	177.4 ± 98.4	5.4
CNA 2	183.5 ± 3.7	170.6 ± 5.6	14.9
CNA 3	109.4 ± 3.3	98.3 ± 15.5	14.1
